# Analysis of Network Pharmacological Efficacy and Therapeutic Effectiveness in Animal Models for Functional Dyspepsia of *Foeniculi fructus*

**DOI:** 10.3390/nu15122644

**Published:** 2023-06-06

**Authors:** Na-Ri Choi, Daehwa Jung, Sang-Chan Kim, Jae-Woo Park, Woo-Gyun Choi, Byung-Joo Kim

**Affiliations:** 1 Department of Longevity and Biofunctional Medicine, Pusan National University School of Korean Medicine, Yangsan 50612, Republic of Korea; nariring@gmail.com; 2 Department of Pharmaceutical Engineering, Daegu Hanny University, Gyeongsan 38610, Republic of Korea; jdh8024@daum.net; 3 College of Oriental Medicine, Daegu Hanny University, Gyeongsan 38610, Republic of Korea; sckim@dhu.ac.kr; 4 Department of Gastroenterology, College of Korean Medicine, Kyung Hee University, Seoul 02447, Republic of Korea; pjw2907@khu.ac.kr; 5 Department of Clinical Korean Medicine, Graduate School of Kyung Hee University, Seoul 02447, Republic of Korea

**Keywords:** *Foeniculi fructus*, functional dyspepsia, network pharmacology, traditional medicine, TCMSP

## Abstract

For centuries, *Foeniculi fructus* (*F. fructus*) has been used as a traditional herbal medicine in China and Europe and is widely used as a natural therapy for digestive disorders, including indigestion, flatulence, and bloating. The mechanism of *F. fructus* that alleviates functional dyspepsia was analyzed through network pharmacology, and its therapeutic effect on an animal model of functional dyspepsia were investigated. The traditional Chinese medicine systems pharmacology (TCMSP) database was used to investigate the compounds, targets, and associated diseases of *F. fructus*. Information on the target genes was classified using the UniProtdatabase. Using the Cytoscape 3.9.1 software, a network was constructed, and the Cytoscape string application was employed to examine genes associated with functional dyspepsia. The efficacy of *F. fructus* on functional dyspepsia was confirmed by treatment with its extract in a mouse model of loperamide-induced functional dyspepsia. Seven compounds targeted twelve functional dyspepsia-associated genes. When compared to the control group, *F. fructus* exhibited significant suppression of symptoms in a mouse model of functional dyspepsia. The results of our animal studies indicated a close association between the mechanism of action of *F. fructus* and gastrointestinal motility. Based on animal experimental results, the results showed that *F. fructus* provided a potential means to treat functional dyspepsia, suggesting that its medical mechanism for functional dyspepsia could be described by the relationship between seven key compounds of *F. fructus*, including oleic acid, β-sitosterol, and 12 functional dyspepsia-related genes.

## 1. Introduction

Functional dyspepsia is characterized as a clinical syndrome where individuals experience recurring or persistent discomfort or pain in the upper abdomen, without any identifiable organic disorders underlying the symptoms [1]. In patients with functional dyspepsia, antagonists of histamine H_2_ receptors [2], inhibitors of proton pumps [3], or eradication with *Helicobacter pylori* have shown limited benefits [4], and the outcomes of controlled trials were generally unsatisfactory. Additionally, pharmacological agents (such as cisapride), despite their limited effectiveness, pose the risk of potential side effects such as arrhythmia, cardiovascular disease, headaches, and abdominal pain.

One attractive alternative through a natural approach is the use of herbal remedies, which are recognized to have a low risk of side effects. However, few rigorous clinical studies are available because of the insufficient standardization of herbal ingredients.

*Foeniculi fructus* (*F. fructus*; *Foeniculum vulgare* or fennel) is an umbelliferous plant that is indigenous to southern Europe and the Mediterranean region. It has a long history of traditional herbal medicine use in both China and Europe, dating back to ancient times [5,6,7,8,9,10,11,12,13,14]. This herb has been employed as a natural remedy for various digestive ailments, such as flatulence, bloating, and indigestion. Additionally, it possesses antipyretic, analgesic, and antioxidant properties [5,6,7]. *F. fructus* provides relief from symptoms associated with female menopausal syndrome, helps regulate menstruation, and enhances libido [8]. It also has galactagogue and emmenagogue properties [9]. *F. fructus* has hepatoprotective effects and may be used in pediatric colic [10,11]. In addition, it also functions as a 5-lipoxygenase inhibitor and is known to be effective in suppressing vomiting, gastrointestinal diseases, and anti-allergies [12]. Additionally, in traditional Turkish medicine, *F. fructus* is used as a diuretic, laxative, antispasmodic, lactating stimulant, and a wound dressing [13]. Although few clinical studies have been conducted on fennel, a clinical study in China reported that drinking *F. fructus* tea after open surgery on gynecological malignancies increased intestinal motility, reducing hospitalization periods and complications [14].

**Figure 1:**
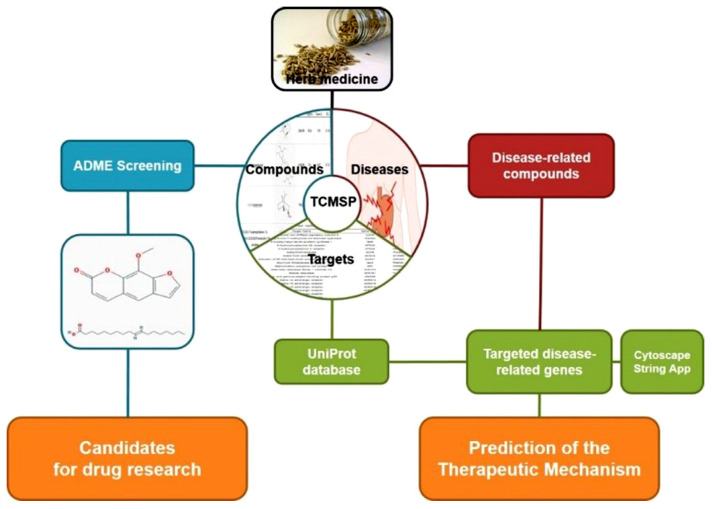
The study protocol schematic.

Conventional biological experimental methodologies frequently face limitations when studying the comprehensive mechanisms of action of traditional herbal medicines, owing to their intricate pharmacological properties [15,16,17,18,19,20,21,22]. To solve these difficulties, network pharmacology, an integrated research field using physics, mathematics, medicine, pharmacology, network science, and computational systems biology, is a new and effective approach [15,16,17,18,19,20,21,22]. The objective of this integrative scientific approach is to elucidate the interactions among biological components such as organs, tissues, cells, proteins, and genes, with the aim of identifying the mechanisms of drug activity and disease pathogenesis [15,16,17,18,19,20,21,22]. To date, network pharmacology studies have identified distinct system-level pharmacological effects, active compounds, and key therapeutic targets, as well as mechanisms (e.g., apoptosis, proliferation, oxidation, and by further confirming the therapeutic regulation of biological processes such as reduction, cell cycle regulation, insulin metabolism, and inflammation) and the multipharmacological properties of traditional herbal drugs exerted by synergistic interactions between multiple compounds and targets [15,16,17,18,19,20,21,22,23,24,25,26,27,28,29,30,31,32,33]. Figure 1 depicts a schematic representation of the study protocol. The objective of our network pharmacology study was to comprehensively understand the impact of *F. fructus* on the molecular mechanisms related to its digestive properties, taking a systems perspective.

## 2. Materials and Methods

### 2.1. Analysis of Network Pharmacology

#### 2.1.1. Identifying Compounds of *F. fructus*

The traditional Chinese medicine systems pharmacology (TCMSP) database was utilized to identify the potentially active compounds in *F. fructus*. We entered ‘*Foeniculi fructus*’ as a search term for herbs.

#### 2.1.2. Target Network

The target information was acquired through the utilization of TCMSP [34]. To associate target proteins with official gene names, the UniProtKB database (https://www.uniprot.org/uniprot, accessed on 7 February 2023) was employed [35].

#### 2.1.3. Analysis of Network

To construct the compound-target network, we utilized Cytoscape 3.9.1 (https://cytoscape.org, accessed on 23 February 2023) [36]. Functional-dyspepsia-associated genes were collected using Cytoscape App., which organized and updated the data weekly [37].

#### 2.1.4. Screening of Active Compound

Physiologically active compounds in *F. fructus* were subjected to screening based on specific criteria related to ADME (absorption, distribution, metabolism, and excretion) parameters. These criteria included MW (molecular weight), OB (oral bioavailability), Caco-2 permeability, and DL (drug similarity). The screening criteria used were as follows: OB ≥ 30%, DL ≥ 0.10, and Caco-2 ≥ −0.4. The compounds that fulfilled the specified criteria were chosen as the active compounds.

### 2.2. Analysis of F. fructus

#### 2.2.1. Instrument and Reagent

A Waters ACQUITY ultra-performance LC system (USA) was utilized to conduct the ultra-performance liquid chromatography (UPLC). A Waters ACQUITY^TM^ photodiode array detector (PDA) and HPLC column (Waters ACQUITY^TM^ BEH C_18_ columns, 1.7 µm, 2.1 × 100), along with the software Empower, were employed for the analysis. The experiment involved the use of methanol (HPLC grade, Junsei, Tokyo, Japan), acetonitrile (HPLC grade, JT-BAKER, Radnor, PA, USA), and tertiary distilled water as reagents. The standard preparations of this experiment were obtained from Anethole (Sigma-Aldrich, St. Louis, MO, USA), R-(a)-phellandrene (Sigma-Aldrich, St. Louis, MO, USA), and 4-Methoxybenzoic acid (ChemFaces, Wuhan, China).

#### 2.2.2. Preparation of the Standard Solution

An accurate measurement of Anethole, R-(a)-phellandrene, and 4-Methoxybenzoic acid was conducted, followed by their dissolution in dimethyl sulfoxide (DMSO) and methanol. Subsequently, a standard undiluted solution was prepared, containing 1 mg per ml of the compounds. In succession, the standard undiluted solution was diluted with methanol to 12.5, 25, 50, and 100 μg per mL, and they were used as standard solutions. All standard materials exhibited determination coefficient (R_2_) values exceeding 0.999 when establishing a standard curve.

#### 2.2.3. Preparation of the Test Liquid for Quantitative Analysis

To perform quantitative analysis, the sample was thoroughly mixed with the test liquid, and precisely 0.2 g of the resulting mixture was added to 10 mL of ethyl alcohol. Subsequently, the mixture was subjected to microwave extraction for a duration of one hour. The resulting test liquid was then filtered using a 0.22 μm membrane filter.

#### 2.2.4. Quantitation of the *F. fructus* Extract

**Figure 2:**
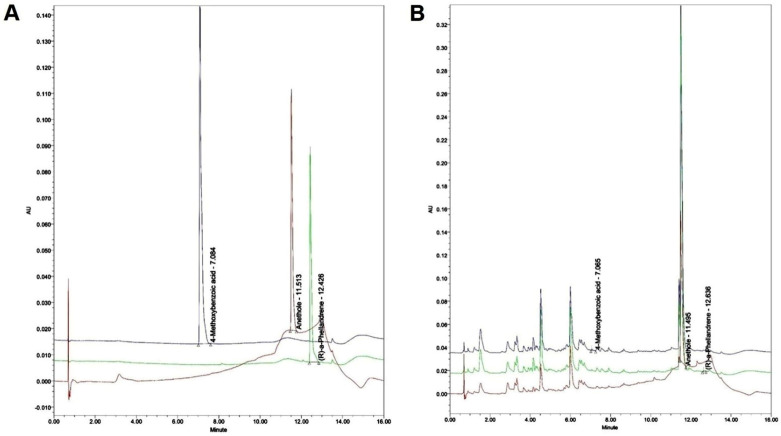
UPLC profiles of three major compounds identified in *F. fructus*. (**A**) UPLC profile of the commercial standard compounds. (**B**) UPLC profile of three major compounds in *F. fructus*. 4-Methoxybenzoic acid and R-(a)-phellandrene were analyzed at 330 nm, and the anethole was analyzed at 306 nm. Black line: 4-Methoxybenzoic acid. Green line: R-(a)-phellandrene. Red line: Anethole.

Ultra-performance liquid chromatography (UPLC) was conducted using a Waters ACQUITY^TM^ ultra-performance LC system (USA) and a Waters ACQUITY^TM^ BEH C_18_ column (1.7 μm, 2.1 × 100). The temperature of the column was kept at room temperature. In PDA analysis, 4-Methoxybenzoic acid and R-(a)-phellandrene were examined at 330 nm, whereas anethole was analyzed at 306 nm (Table 1). The mobile phase used in the analysis consisted of a blend of acetonitrile and water with 0.1% formic acid. The analysis parameters were set as follows: a 2 μL sample injection and a flow rate of 0.4 mL/min. The qualitative analysis was conducted by verifying the retention time, followed by quantitation using the peak area method. The *F. fructus* samples were deposited at the College of Korean Medicine, Daegu Hanny University (Table 2; Figure 2).

### 2.3. Animal Testing

#### 2.3.1. Design of Animal Experiment

A commercial animal breeder (Samtako, Gyeonggi, Republic of Korea) provided a total of 108 specific pathogen-free (SPF) ICR mice. These mice were all male, weighed between 19–21 g, and were five weeks old at the time of purchase. The mice were housed in a temperature-controlled room within a specific pathogen-free (SPF) facility, where the temperature was maintained at 22 ± 2 °C and the relative humidity at 60 ± 5%. The mice followed a 12/12 h light/dark cycle in their housing environment. The mice were provided with unlimited access to commercial standard chow (Samtako, Gyeonggi, Republic of Korea) and tap water. Following a one-week acclimatization period, the mice were randomly divided into three experimental groups: the first group for small intestine motility (6 mice/group, *n* = 36), the second group for gastric emptying test (6 mice/group, *n* = 36), and the third group for Western blot, qPCR, and histopathology (3 mice/group, *n* = 18). The sets were divided into six groups: control, loperamide (negative control, 10 mg/kg), three different doses of *F. fructus* (25, 50, and 100 mg/kg), and mosapride (positive control, 3 mg/kg). In general, the treatment dose of mosapride was 3.1 mg/kg in mice [38]. Distilled water was used to prepare *Foeniculi fructus* and mosapride. Each group received oral administration of either distilled water (control and loperamide groups), *F. fructus*, or mosapride for a duration of three consecutive days [39,40]. The experiments and animal care procedures followed the guidelines provided by the Animal Care and Use Committee of the Pusan National University Animal Research Institute (PNU-2022-0160) and the regulations outlined in the guidelines for the management and utilization of laboratory animals at the US National Institutes of Health.

#### 2.3.2. Assessment of Gastric Weight and Gastric Emptying

The mice underwent a 19 h fasting period with unrestricted access to tap water. The volume of phenol red solution (500 µL) and the 50% delayed gastric emptying time point was based on previously established study protocols [41,42]. After a 30 min period following the administration of 0.05% phenol red (dyeing substance that checks the level of gastric emptying), the mice were humanely euthanized. The stomachs were promptly excised and weighed. Subsequently, the stomachs were treated with 5 mL of 0.1 N sodium hydroxide solution to measure the optical density of residual phenol red. Additionally, 0.5 mL of trichloroacetic acid (20% *w*/*v*) was added to the stomachs. The produced homogenate was centrifuged at 3000 rpm for 20 min and then mixed with 0.5 N sodium hydroxide solution with supernatant 1 milliliter. In addition, the optical density was measured at a wavelength of 560 nm with a spectrophotometer.

The emission values mentioned above were derived using the following formula:

gastric emptying (%) = (1 − X/Y) × 100

X: Optical density of the phenol red remaining on it. Y: Optical density of the phenol red mixture with sodium hydroxide under test tube conditions.

#### 2.3.3. Assessment of Intestinal Transit Rate by Evans Blue

To measure the intestinal transit rate, the Evans blue diet method was used, in which 5% Evans blue (dyeing substance that checks the level of intestinal transit rate) was prepared in distilled water, as previously described [43]. Evans blue diet was orally administered (250 μL/20 g mouse) 30 min after IP injection of loperamide. After a 30 min period following the administration of the Evans blue dye, the mice were humanely euthanized. The distance covered by the Evans blue dye within the small intestine, specifically from the pylorus to the cecum, was measured to determine the intestinal transit distance. The above time points were selected as per the methods of an earlier study [44].

#### 2.3.4. Western Blot Analysis for Check of Protein Level

To measure the gastric protein levels of neuronal nitric oxide synthase (nNOS), TEME16A, and TRPM7, gastric tissues were homogenized in RIPA lysis buffer. Following a 5 min denaturation period by boiling, the proteins were subjected to electrophoresis on a 10% polyacrylamide gel and subsequently transferred to a nitrocellulose (NC) membrane. After blocking in 5% skim milk for 30 min, membranes were tested overnight at 4 °C with nNOS (1:1000, ab76067), TEME16A (1:1000, a72984), TRPM7 (1:200, ab135817), or β-actin (1:5000, sc-47778). After washing the membranes, they were incubated with a horseradish peroxidase (HRP)-conjugated rabbit antibody (diluted 1:5000 against nNOS, TEME16A, and TRPM7) or an HRP-conjugated mouse antibody (diluted 1:5000 against β-actin) for a duration of 1 h at room temperature. Protein visualization was performed using Western Bright Sirius (Advansta, San Jose, CA, USA), and protein expression was examined using an ImageQuant LAS 4000 system (GE Healthcare, Chicago, IL, USA). Protein expression quantification was conducted using ImageJ software (NIH).

#### 2.3.5. Quantitative Real-Time PCR to Evaluate Gene Expression

In order to determine the expression of genes related to muscle contraction, including anoctamin-1 (ANO1), ryanodine receptor 3 (RYR3), smooth muscle cell myosin light chain kinase (smMLCK), and 5HT4 receptor (5HT4R), total mRNA was extracted from the stomach tissues using Trizol reagents (Invitrogen, Waltham, MA, USA). cDNA reverse transcription kit (M-MLV Reverse Transcriptase, Promega, Madison, WI, USA) was utilized to synthesize cDNA from the entire RNA sample (1 μg). For qPCR analysis, iTaq Universal SYBR Green Supermix (Bio-Rad, Hercules, CA, USA) was employed along with the primers provided in Table 3. The StepOnePlus Real-Time PCR System (Applied Biosystems, Foster City, CA, USA) was utilized for the analysis of gene expression data.

## 3. Results

### 3.1. Target Information Derived by Examining Correlations between Compounds and Targets

**Figure 3:**
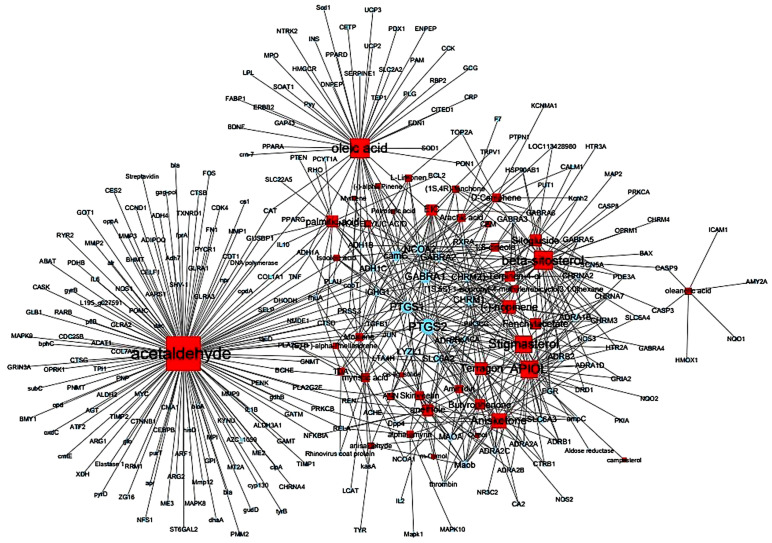
Compound-target network of *F. fructus*. The node size depends on the number of connected edges. The compound is represented as a red square-shaped node, and the targets are represented as a blue round-shaped node.

A total of 45 potentially active compounds in *F. fructus* were identified with the TCMSP database (Supplementary Materials Table S1). Out of the identified compounds, 41 exhibited information regarding their targets (Supplementary Materials Table S2). These 41 compounds interacted with a total of 260 targets, involving a combination of 611 components. As shown in Figure 3, acetaldehyde was linked to the greatest number of targets (142 genes), followed by oleic acid (48 genes), β-sitosterol (38 genes), APIOL (31 genes), stigmasterol (31 genes), anisketone (24 genes), (−)-nopinene (21 genes), and terragon (20 genes).

### 3.2. A Total of Nine Compounds Met ADME Requirements for Active Compounds

A total of nine compounds satisfied the screening criteria for active compounds (Table 4): ammidin, β-sitosterol, EIC, oleic acid, majudin, oleic acid, petroselic acid, stigmasterol, and uvadex.

### 3.3. Thirty-Two Compounds Associated with Gastrointestinal (GI) Diseases Were Identified in F. fructus

**Figure 4:**
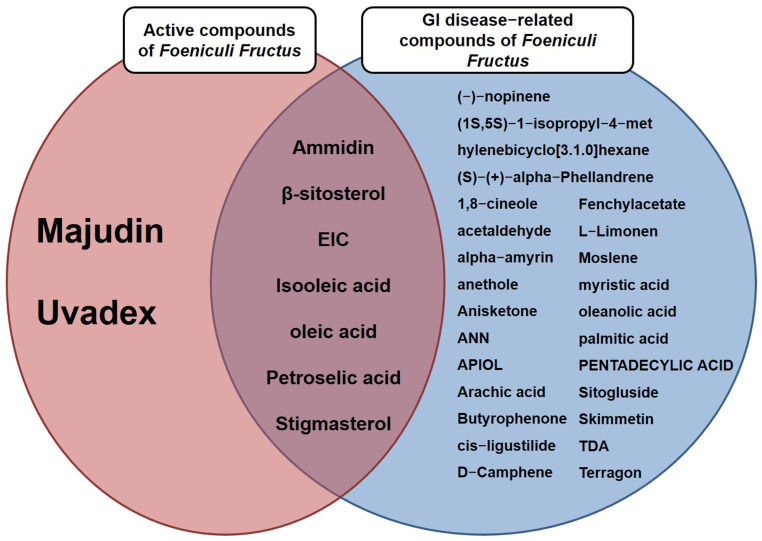
The Venn diagram of bioactive compounds of *F. fructus* related to GI disease.

Furthermore, we utilized the TCMSP database to establish relationships between compounds, targets, and diseases. It was observed that 32 compounds were linked to gastrointestinal (GI) diseases (Table 5). In particular, ammidin, β-sitosterol, EIC, isooleic acid, oleic acid, petroselic acid, and stigmasterol were related with gastrointestinal diseases. Other compounds associated with gastrointestinal diseases, including (−)-nopinene, (1*S*,5*S*)-1-isopropyl-4-methylenebicyclo [3.1.0]hexane, (S)-(+)-alpha-phellandrene, 1,8-cineole, acetaldehyde, alpha-amyrin, anethole, anisketone, ANN, APIOL, arachic acid, butyrophenone, cis-ligustilide, d-camphene, fenchylacetate, l-limonen, moslene, myristic acid, oleanolic acid, palmitic acid, pentadecylic acid, sitogluside, skimmetin, TDA, and terragon were confirmed as non-active compounds (Figure 4).

### 3.4. All 31 GI Disease-Related Compounds in F. fructus except Oleanolic Acid Were Associated with Functional Dyspepsia

**Figure 5:**
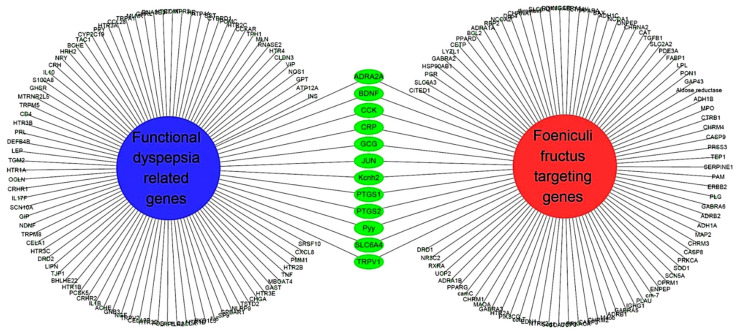
Network of functional dyspepsia related genes and *F. fructus* target genes. There were 12 genes in common in two places.

To investigate the relationship between *F. fructus* and functional dyspepsia, we used the Cytoscape App to determine genetic information related to functional dyspepsia. With a score cutoff 0.40 and a maximum of 100 proteins, we initially identified 100 genes associated with functional dyspepsia (Supplementary Materials Table S3). Based on the obtained results, we constructed a network comprising functional dyspepsia-related genes and the target genes of activated compounds in *F. fructus* (Figure 5). Fourteen genes corresponding to two gene sets were identified, and the functional dyspepsia-related genes targeted by the activated *F. fructus* compound were ADRA2A, BDNF, CCK, CRP, GCG, JUN, Kcnh2, PTGS1, PTGS2, Pyy, SLC6A4, and TRPV.

### 3.5. Network of Functional Dyspepsia-Associated Genes and Compounds

**Figure 6:**
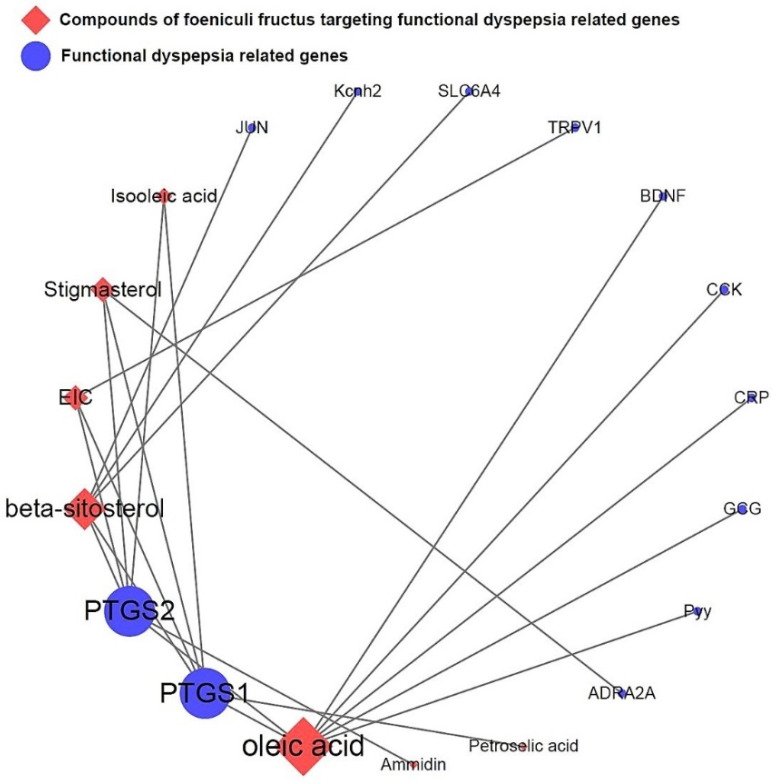
Network of compounds of *F. fructus* and functional dyspepsia-related genes.

The network depicted in Figure 6 illustrates the relationship between activated compounds in *F. fructus* and target genes associated with functional dyspepsia. Notably, PTGS1 and PTGS2 exhibited the strongest associations with functional dyspepsia. In summary, ammidin, EIC, oleic acid, petroselic acid, stigmasterol, β-sitosterol, and oleic acid were active compounds that targeted functional dyspepsia-associated genes, suggesting that they could be potential drug candidates.

### 3.6. Mouse Experiment on Delayed Gastric Emptying

**Figure 7:**
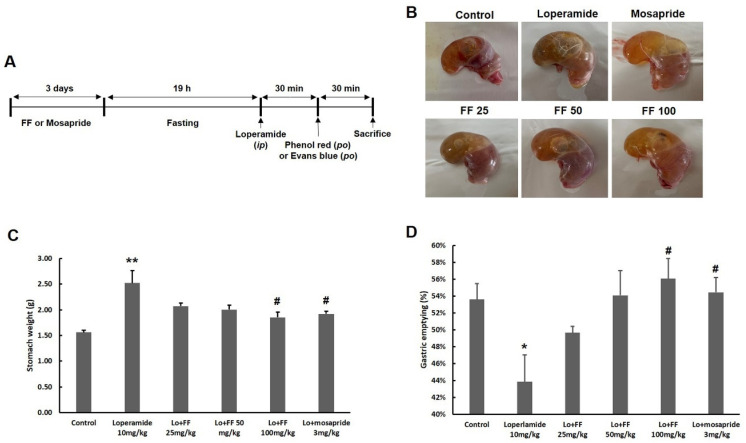
Results of *F. fructus* (FF) on gastric emptying. The experimental schedule is summarized in (**A**). For 3 days, mice (*n* = 6/group) were treated by po with 25, 50, and 100 mg/kg of FF or 3 mg/kg of mosapride and then treated by IP injection with 10 mg/kg of loperamide. After the treatment of phenol red, results of visualization (**B**), weight of stomach (**C**), and results of gastric emptying (**D**) are presented. The data are organized as the mean ± SEM. * *p* < 0.05, ** *p* < 0.01 for the Control group; # *p* < 0.05 for the loperamide group.

Loperamide injection induced gastric food retention, whereas pretreatment with *F. fructus* decreased this effect, as seen by macroscopic observation (Figure 7B). This finding was validated using quantitative analysis. The group treated with *F. fructus* exhibited a significantly lower gastric weight compared to the loperamide group (*p* < 0.05, as shown in Figure 7C). The pretreatment with *F. fructus* resulted in a significant reduction in the amount of phenol red retention in the stomach compared to the loperamide group (*p* < 0.05, as depicted in Figure 7D). Pretreatment with mosapride also had similar effects as the *F. fructus* treatment.

### 3.7. Mouse Experiment on Molecules Involved in Gastrointestinal Motility

**Figure 8:**
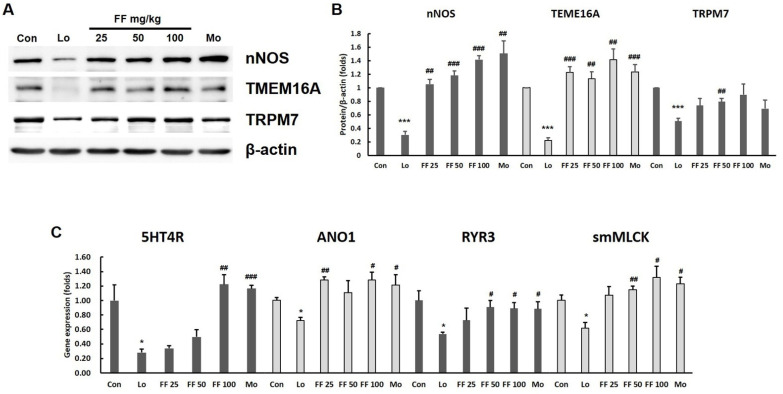
Results of *F. fructus* (FF) on GI motility-associated molecules in stomach tissue. The analyses of Western blot for nNOS, TMEM16A, and TRPM7 (**A**) and semi-quantifications (**B**) were conducted (*n* = 3). The analyses of mRNA expression of GI motility-associated genes were performed (**C**) (*n* = 3) in the stomach tissue. The data are organized as the mean ± SEM. * *p* < 0.05, *** *p* < 0.001 for the Control group; # *p* < 0.05, ## *p* < 0.01, ### *p* < 0.001 for the loperamide group.

Loperamide injection sharply attenuated nNOS, TEME16A, and TRPM7 protein expression in gastric tissue, whereas pretreatment with *F. fructus* sharply increased nNOS, TEME16A, and TRPM7 protein expression (*p* < 0.01, Figure 8A,B). Loperamide injection also decreased smooth-muscle-contraction-related gene expression, including 5HT4R, RYR3, ANO1, and smMLCK. These changes were inhibited by pretreatment with *F. fructus* (*p* < 0.05, *p* < 0.01, Figure 8C). Mosapride had a positive effect on nNOS, TEME16A, and TRPM7 proteins and 5HT4R, RYR3, ANO1, and smMLCK gene expression.

### 3.8. Mouse Experiment on Intestinal Motility

**Figure 9:**
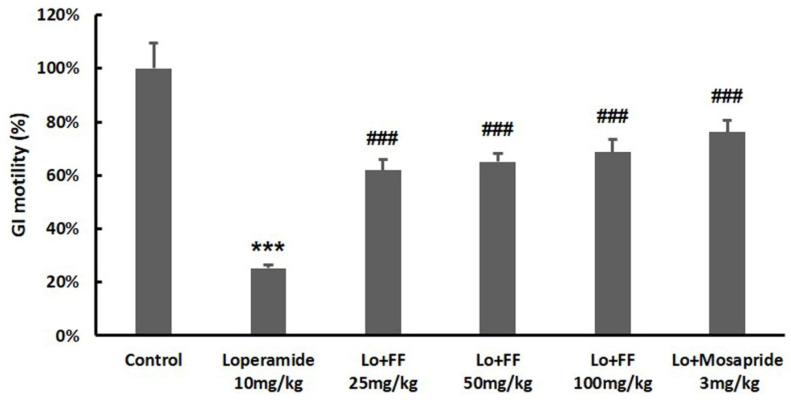
Results of *F. fructus* (FF) on small intestinal motility. For 3 days, mice (*n* = 6/group) were treated by po with 25, 50, and 100 mg/kg of FF or 3 mg/kg of mosapride and then treated by IP injection with 10 mg/kg of loperamide. After 30 min of treatment with Evans blue, the distances stained were checked and quantified. The data are organized as the mean ± SEM. *** *p* < 0.001 for the Control group; ### *p* < 0.001 for the loperamide group.

The administration of loperamide significantly decreased small intestine motility when compared to the control group. This suppression of small intestine motility was significantly restored by pretreatment with *F. fructus* (*p* < 0.01; Figure 9). Pretreatment with mosapride also sharply restored the motility of the small intestine, similar to that of *F. fructus.*

## 4. Discussion

For centuries, *F. fructus* has served as a renowned traditional herbal medicine in China and Europe, with extensive cultivation in southern Europe and the Mediterranean region. Multiple studies have demonstrated the antitumor, antioxidant, cytoprotective, hypoglycemic, hepatoprotective, and estrogenic properties of *F. fructus* [11,45,46,47,48]. Furthermore, it has proven effective in managing various infectious disorders caused by bacteria, fungi, mycobacteria, protozoa, and viruses [49,50,51]. The seeds of *F. fructus* are known to be associated with menstrual control and alleviation of symptoms of female menopausal syndrome [8], and the aqueous extract of *F. fructus* has a significant antiulcer effect against ethanol-induced gastric lesions [52]. In addition, the essential oil of *F. fructus* regulates intestinal smooth muscle motility and reduces intestinal gas. It is also used in the treatment of spasmodic gastrointestinal disorders and indigestion caused by gastrointestinal disorders along with other plant medicines [53]. However, this has not yet been studied.

To uncover the bioactive components and therapeutic mechanisms of *F. fructus*, a comprehensive approach combining network-based pharmacological analysis and experimental validation was employed in this study. The investigation yielded the identification of 45 compounds, among which 9 were found to be active compounds (Supplementary Materials Table S1). Additionally, target information was available for 41 out of the 45 compounds (Supplementary Materials Table S2), resulting in the identification of 260 target genes (Supplementary Materials Table S3). FD- and *F. fructus*-related genes included alpha-2A adrenergic receptor (ADRA2A), brain-derived neurotrophic factor (BDNF), cholecystokinin (CCK), C-reactive protein (CRP), glucagon (GCG), transcription factor Jun (JUN), hERG (Kcnh2), cyclooxygenase 1 (PTGS1), cyclooxygenase 2 (PTGS2), peptide YY (Pyy), transient receptor potential cation channel subfamily V member 1 (TRPV1), and serotonin transporter (SLC6A4) (Figure 5). The findings were in accordance with prior research studies. Particularly, as depicted in Figure 6, PTGS1 and PTGS2 were the targets of most of the activated FD-related compounds in *F. fructus*, suggesting that the compounds in *F. fructus* could synergistically modulate the levels of PTGS1 and PTGS2. PTGS1 was associated with dyspepsia and chronic cystitis [54] and contributed to the maintenance of the mucus barrier and mucosal blood flow in the stomach [55]. The involvement of PTGS2 was crucial in several key aspects of mucosal defense, making a substantial contribution to the resolution of gastroenteritis and playing a significant role in the regulation of ulcer healing. PTGS2 also contributed to long-term changes in gastrointestinal function following inflammation [56]. These results indicated that the effects of *F. fructus* PTGS1 and PTGS2 on the treatment mechanism of functional dyspepsia were related.

Functional dyspepsia-related active compounds including ammidin, EIC, oleic acid, petroselic acid, stigmasterol, β-sitosterol, and oleic acid were identified (Figure 6). Six compounds were found to target PTGS1 and PTGS2, and oleic acid targeted BDNF, CRP, CCK, GCG, PTGS1, PTGS2, and Pyy. β-sitosterol targeted JUN, Kcnh2, PTGS1, PTGS2, and SLC6A4. The association between major compounds and functional dyspepsia has been verified by multiple studies. The presence of oleic acid in emulsions triggers a nutrient-induced negative feedback mechanism within the small intestine. This mechanism effectively decelerates gastrointestinal transit and alleviates symptoms of diarrhea [57]. In mice, β-Sitosterol enhances antibacterial activity and effectively mitigates DSS-induced colitis [58].

Figure 7 demonstrated the multi-component multi-targeting attributes of herbal medicines, revealing their interaction with an average of around 15 target genes. The synergistic effects of various compounds found in *F. fructus* predicted its potential as a therapeutic agent for functional dyspepsia. In this study, we examined the therapeutic effects of *F. fructus* using a mouse model of functional dyspepsia. Our results showed that *F. fructus* has therapeutic potential for functional dyspepsia. In addition, it was found that there was a therapeutic effect on functional dyspepsia through a mechanism related to the interaction between seven major active ingredients of *F. fructus*, such as oleic acid and β-sitosterol, and 12 functional dyspepsia-related genes, including PTGS1 and PTGS2.

There are about 100 trillion microorganisms in the human gut [59]. The largest number of bacteria in the population of more than 100 species are Gram-positive *Firmicutes* that produce short-chain fatty acids and Gram-negative *Bacteroidetes* that produce hydrogen, *Proteobacteria*, and *Actionobacteria* [60]. This rich and diverse microbial ecosystem acts as an effective barrier to pathogens, interacts with the immune system, and represents a key factor in maintaining host homeostasis [61]. Many studies have shown that the occurrence of gastrointestinal diseases is caused by qualitative or quantitative changes in the composition of gut microbiota [62,63]. Functional dyspepsia mentioned in this paper is also known to be caused by gut microbiota [64,65] and thus is becoming our new treatment approach to functional dyspepsia. *F. fructus* is not well known for its regulation of gut microbiota, and there are few studies. In the future, it is thought that research on the control of gut microbiota by *F. fructus* may be needed.

We selected a functional dyspepsia animal model using loperamide to test the pharmacological effects of *F. fructus* and to identify the mechanism of action. Loperamide, an agonist of the μ-opioid receptor, is used to trigger dyspepsia [66].

The administration of loperamide via injection resulted in a delay in gastric emptying, as evidenced by the presence of postprandial satiety, an increase in gastric weight, and the retention of phenol red in the stomach. Pretreatment with *F. fructus* significantly prevented the delay in gastric emptying. (Figure 7). In the majority of clinical studies, delayed gastric emptying has been consistently observed as a characteristic feature of functional dyspepsia [67,68]. In our model, treatment with loperamide resulted in a significant reduction in GI motility (Figure 8). Overlap between functional dyspepsia and irritable bowel syndrome (IBS) has been reported in previous studies, with a substantial degree of concurrence estimated at approximately 19% [69,70]. Postprandial satiety is a major complaint in patients with IBS and FD, in which constipation predominates [71]. The delay in GI immobility induced by loperamide was significantly alleviated by pretreatment with *F. fructus* extract (Figure 9).

To elucidate the mechanism for the therapeutic effect of *F. fructus*, the protein level of nNOS and the expressions of four genes (5-HT4R, RYR3, ANO1, and smMLCK) were confirmed in the gastric tissue. NO produced by nNOS, a well-known neurotransmitter in the gastrointestinal tract, plays an important role in smooth muscle cell relaxation [72]. Furthermore, the presence of nNOS gene polymorphisms has been linked to an increased susceptibility to functional dyspepsia (FD) and the development of postprandial discomfort and epigastric pain [73]. Notably, pretreatment with *F. fructus* effectively mitigated the reduction in nNOS protein levels induced by loperamide while also leading to an elevation in the expression of smooth muscle contraction-related genes such as 5-HT4R, RYR3, ANO1, and smMLCK (Figure 8). The activation of intracellular calcium efflux into interstitial cells of Cajal (ICC) and the generation of slow waves both rely on the functioning of ANO1 [74,75]. The Ca^2+^ spark creates a slow wave and is regulated by ANO1 in the membrane of ICC and by RYR3 molecules in the endoplasmic reticulum [76,77]. A decrease in smMLCK activity in the smooth muscle in intestinal motility disorders is characterized by diminished peristalsis [78]. ANO1 and smMLCK were downregulated in an animal model of diabetic gastroparesis [79,80]. The findings implied that *F. fructus* exhibited pharmacological activity in the modulation of interstitial cells of Cajal (ICC) within the gastrointestinal tract. By modulating nNOS, *F. fructus* could restore normal peristalsis and activate contraction-related molecules.

## 5. Conclusions

Through the utilization of network-based pharmacological analysis, it was determined that seven compounds and 12 genes found in *F. fructus* were linked to functional dyspepsia. Our animal studies have shown that *F. fructus* suppressed functional dyspepsia-like symptoms in a mouse model of functional dyspepsia. The findings of this study indicated that *F. fructus* possesses therapeutic potential for treating functional dyspepsia.

## Figures and Tables

**Table 1:** The analysis condition of 4-Methoxybenzoic acid, Anethole, and R-(α)-Phellandrene.

Time (min)	0.1% FA/Water (%)	0.1% FA/Acetonitrile (%)	Flow Rate (mL/min)
0	98	2	0.40
1.0	98	2	0.40
3.0	85	15	0.40
5.0	75	25	0.40
6.0	55	45	0.40
8.0	50	50	0.40
9.0	30	70	0.40
10.0	10	90	0.40
12.0	2	98	0.40
14.0	98	2	0.40
16.0	98	2	0.40

**Table 2:** Contents of the *F. fructus* marker compounds by UPLC.

*F. fructus* (Unit: mg/kg)	
4-Methoxybenzoic acid	0.219 ± 0.042
Anethole	63.029 ± 2.076
R-(a)-Phellandrene	0.792 ± 0.059

**Table 3:** Summary for gene sequence.

Gene	Primer	Sequence (5′ to 3′)	Product Length (bp)
5HT4R	Forward	AGTTCCAACGAGGGTTTCAGG	92
	Reverse	CAGCAGGTTGCCCAAGATG	
ANO1	Forward	GGCATTTGTCATTGTCTTCCAG	140
	Reverse	TCCTCACGCATAAACAGCTC	
RYR3	Forward	GGCCAAGAACATCAGAGTGACTAA	79
	Reverse	TCACTTCTGCCCTGTCAGTTTC	
smMLCK	Forward	AGAAGTCAAGGAGGTAAAGAATGATGT	76
	Reverse	CGGGTCGCTTTTCATTGC	
GAPDH	Forward	CATGGCCTTCCGTGTTCCT	103
	Reverse	CCTGCTTCACCACCTTCTTGA	

**Table 4:** Active compounds of *F. fructus*.

Molecule Name	Structure	MW	OB(%)	Caco-2	DL
Ammidin	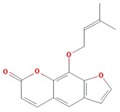	270.3	34.55	1.13	0.22
beta-sitosterol	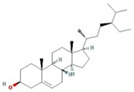	414.79	36.91	1.32	0.75
EIC	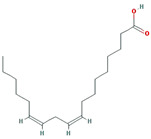	280.5	41.9	1.16	0.14
Isooleic acid	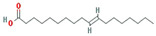	282.52	33.13	1.15	0.14
Majudin	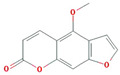	216.2	42.21	0.94	0.13
oleic acid	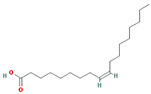	282.52	33.13	1.17	0.14
Petroselic acid	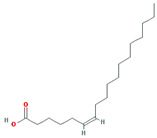	282.52	33.13	1.17	0.14
Stigmasterol	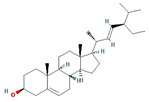	412.77	43.83	1.44	0.76
Uvadex	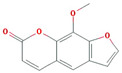	216.2	35.3	1.05	0.13

**Table 5:** Compounds and targets related to GI diseases.

Molecule Name	Gene Name	Disease Name
(−)-nopinene	PTGS1	* Functional dyspepsia
	PTGS2	* Functional dyspepsia Colorectal cancer Peutz–Jeghers syndrome Oropharyngeal squamous cell carcinoma
(1*S*,5*S*)-1-isopropyl-4-methylenebicyclo [3.1.0]hexane	PTGS2	* Functional dyspepsia Colorectal cancer Peutz–Jeghers syndrome Oropharyngeal squamous cell carcinoma
(*S*)-(+)-alpha-Phellandrene	ACHE	* Functional dyspepsia
1,8-cineole	NOS3	Colorectal cancer
	PTGS2	* Functional dyspepsia Colorectal cancer Peutz–Jeghers syndrome Oropharyngeal squamous cell carcinoma
acetaldehyde	BCHE	* Functional dyspepsia
	CTNNB1	Colorectal cancer
	FOS	* Functional dyspepsia
	IL1B	* Functional dyspepsia
	IL6	* Functional dyspepsia
	JUN	* Functional dyspepsia
	LTA4H	Esophageal cancer
	MAPK8	Crohn’s Disease, unspecified
	MAPK9	Crohn’s Disease, unspecified
	MMP1	Kaposi’s Sarcoma Pancreatic Cancer
	Mmp12	Crohn’s Disease, unspecified Gastro-intestinal ulcers Ulcerative colitis
	MMP2	Kaposi’s Sarcoma Pancreatic Cancer
	MMP3	Pancreatic Cancer
	NOS1	* Functional dyspepsia
	OPRK1	Diarrhea
	POMC	* Functional dyspepsia
	PTGS1	* Functional dyspepsia
	PTGS2	* Functional dyspepsia Colorectal cancer Peutz–Jeghers syndrome Oropharyngeal squamous cell carcinoma
	RARB	Pancreatic Cancer
	RRM1	Pancreatic Neoplasms
	TNF	* Functional dyspepsia Crohn’s Disease, unspecified
alpha-amyrin	PTGS2	* Functional dyspepsia Colorectal cancer Peutz–Jeghers syndrome Oropharyngeal squamous cell carcinoma
Ammidin	PTGS2	* Functional dyspepsia Colorectal cancer Peutz–Jeghers syndrome Oropharyngeal squamous cell carcinoma
anethole	JUN	* Functional dyspepsia
Anisketone	ACHE	* Functional dyspepsia
	ADRA2A	* Functional dyspepsia
	CA2	Pancreatic Cancer
	NOS3	Colon cancer
	PTGS1	* Functional dyspepsia
	PTGS2	* Functional dyspepsia Colorectal cancer Peutz–Jeghers syndrome Oropharyngeal squamous cell carcinoma
ANN	PTGS1	* Functional dyspepsia
	PTGS2	* Functional dyspepsia Colorectal cancer Peutz–Jeghers syndrome Oropharyngeal squamous cell carcinoma
APIOL	ADRA2A	* Functional dyspepsia
	LTA4H	Esophageal cancer
	NOS3	Colon cancer
	PTGS1	* Functional dyspepsia
	PTGS2	* Functional dyspepsia Colorectal cancer Peutz–Jeghers syndrome Oropharyngeal squamous cell carcinoma
	SLC6A4	* Functional dyspepsia
Arachic acid	HSP90AB1	Gastrointestinal Stromal Tumors (GIST)
	PTGS1	* Functional dyspepsia
	PTGS2	* Functional dyspepsia Colorectal cancer Peutz–Jeghers syndrome Oropharyngeal squamous cell carcinoma
beta-sitosterol	HSP90AB1	Gastrointestinal Stromal Tumors (GIST)
	JUN	* Functional dyspepsia
	Kcnh2	* Functional dyspepsia
	OPRM1	Diarrhea Opioid-induced bowel dysfunction
	PTGS1	* Functional dyspepsia
	PTGS2	* Functional dyspepsia Colorectal cancer Peutz–Jeghers syndrome Oropharyngeal squamous cell carcinoma
	SLC6A4	* Functional dyspepsia
Butyrophenone	CA2	Pancreatic Cancer
	PTGS1	* Functional dyspepsia
	PTGS2	* Functional dyspepsia Colorectal cancer Peutz–Jeghers syndrome Oropharyngeal squamous cell carcinoma
cis-ligustilide	PTGS2	* Functional dyspepsia Colorectal cancer Peutz–Jeghers syndrome Oropharyngeal squamous cell carcinoma
D-Camphene	Kcnh2	* Functional dyspepsia
	PTGS2	* Functional dyspepsia Colorectal cancer Peutz–Jeghers syndrome Oropharyngeal squamous cell carcinoma
EIC	PTGS1	* Functional dyspepsia
	PTGS2	* Functional dyspepsia Colorectal cancer Peutz–Jeghers syndrome Oropharyngeal squamous cell carcinoma
	TRPV1	* Functional dyspepsia
Fenchylacetate	PTGS2	* Functional dyspepsia Colorectal cancer Peutz–Jeghers syndrome Oropharyngeal squamous cell carcinoma
Isooleic acid	PTGS1	* Functional dyspepsia
	PTGS2	* Functional dyspepsia Colorectal cancer Peutz–Jeghers syndrome Oropharyngeal squamous cell carcinoma
L-Limonen	PTGS2	* Functional dyspepsia Colorectal cancer Peutz–Jeghers syndrome Oropharyngeal squamous cell carcinoma
Moslene	ACHE	* Functional dyspepsia
	PTGS2	* Functional dyspepsia Colorectal cancer Peutz–Jeghers syndrome Oropharyngeal squamous cell carcinoma
myristic acid	BCHE	* Functional dyspepsia
	PTGS1	* Functional dyspepsia
	PTGS2	* Functional dyspepsia Colorectal cancer Peutz–Jeghers syndrome Oropharyngeal squamous cell carcinoma
oleanolic acid	AMY2A	Pancreatic disease
oleic acid	BDNF	* Functional dyspepsia
	CCk	* Functional dyspepsia
	CRP	* Functional dyspepsia
	GCG	* Functional dyspepsia
	PTGS1	* Functional dyspepsia
	PTGS2	* Functional dyspepsia Colorectal cancer Peutz–Jeghers syndrome Oropharyngeal squamous cell carcinoma
	Pyy	* Functional dyspepsia
palmitic acid	IL10	* Functional dyspepsia
	PTGS1	* Functional dyspepsia
	PTGS2	* Functional dyspepsia Colorectal cancer Peutz–Jeghers syndrome Oropharyngeal squamous cell carcinoma
	TNF	* Functional dyspepsia Crohns’s Disease, unspecified
PENTADECYLIC ACID	PTGS1	* Functional dyspepsia
	PTGS2	* Functional dyspepsia Colorectal cancer Peutz–Jeghers syndrome Oropharyngeal squamous cell carcinoma
Petroselic acid	PTGS1	* Functional dyspepsia
Sitogluside	HSP90AB1	Gastrointestinal Stromal Tumors (GIST)
	HTR3A	* Functional dyspepsia Diarrhea Postoperative nausea and vomiting Irritable bowel syndrome Chemotherapy-induced nausea and vomiting
	Kcnh2	* Functional dyspepsia
	PTGS1	* Functional dyspepsia
	PTGS2	* Functional dyspepsia Colorectal cancer Peutz–Jeghers syndrome Oropharyngeal squamous cell carcinoma
Skimmetin	ADRA2A	* Functional dyspepsia
	LTA4H	Esophageal cancer
	PTGS1	* Functional dyspepsia
	PTGS2	* Functional dyspepsia Colorectal cancer Peutz–Jeghers syndrome Oropharyngeal squamous cell carcinoma
Stigmasterol	ADRA2A	* Functional dyspepsia
	LTA4H	Esophageal cancer
	PTGS1	* Functional dyspepsia
	PTGS2	* Functional dyspepsia Colorectal cancer Peutz–Jeghers syndrome Oropharyngeal squamous cell carcinoma
TDA	PTGS1	* Functional dyspepsia
Terragon	ADRA2A	* Functional dyspepsia
	PTGS1	* Functional dyspepsia
	PTGS2	* Functional dyspepsia Colorectal cancer Peutz–Jeghers syndrome Oropharyngeal squamous cell carcinoma

* After utilizing the Cytoscape StringApp to explore the association between *F. fructus* and functional dyspepsia, genes relevant to functional dyspepsia were included in this table.

## Data Availability

The original data are available upon reasonable request to the corresponding author.

## Supplementary Materials

The following supporting information can be downloaded at: https://www.mdpi.com/article/10.3390/nu15122644/s1, Table S1: Potential active compounds of *Foeniculi fructus*. Table S2: Target genes of *Foeniculi fructus*. Table S3: One hundred functional dyspepsia-related genes.
