# Supplementary material for: Analysis of Network Pharmacological Efficacy and Therapeutic Effectiveness in Animal Models for Functional Dyspepsia of Foeniculi fructus
Source: Nutrients. 2023 Jun 6;15(12):2644. doi: 10.3390/nu15122644 (PMC10301275; doi:10.3390/nu15122644)
Supplement: Supplementary file 1 [file nutrients-15-02644-s001.zip › Table S1 Potential active compounds of Foeniculi fructus.pdf]

Supplementary Materials Table S1

Potential active compounds of *Foeniculi Fructus*.

Molecule Name	Structure	MW	OB (%)	Caco-2	DL
(-)-alpha-Pinene	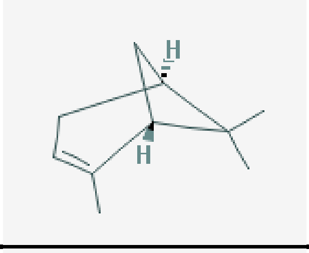	136.26	46.25	1.85	0.05
(-)-nopinene	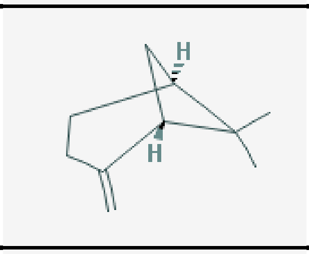	136.26	44.84	1.8	0.05
()-Terpinen-4-ol	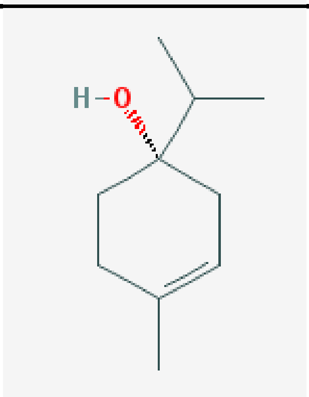	154.28	81.41	1.36	0.03
(1S,4R)-fenchone	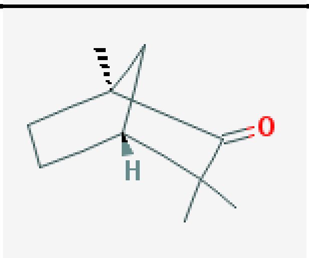	152.26	72.64	1.35	0.05
(1S,5S)-1-isopropyl-4-methylenebicyclo[3.1.0]hexane	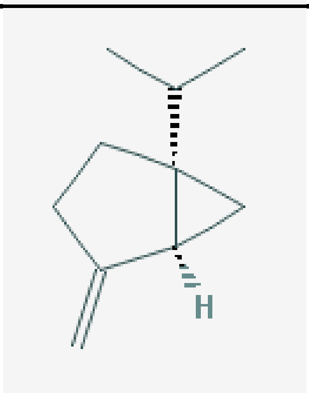	136.26	46.21	1.83	0.04

Molecule Name	Structure	MW	OB (%)	Caco-2	DL
(S)-(+)-alpha-Phellandrene	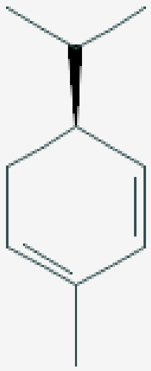	136.26	27.9	1.87	0.02
1,8-cineole	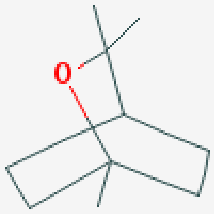	154.28	39.73	1.57	0.05
acetaldehyde	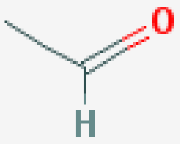	44.06	42.25	1.01	0
alpha-amyrin	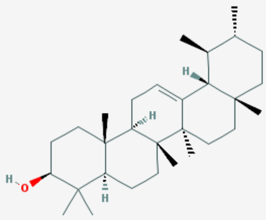	426.8	10.28	1.44	0.76
Ammidin	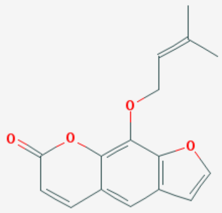	270.3	34.55	1.13	0.22

Molecule Name	Structure	MW	OB (%)	Caco-2	DL
anethole	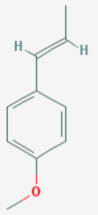	148.22	32.49	1.75	0.03
anisaldehyde	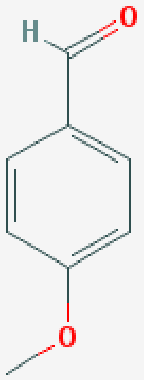	136.16	21.54	1.12	0.02
Anisketone	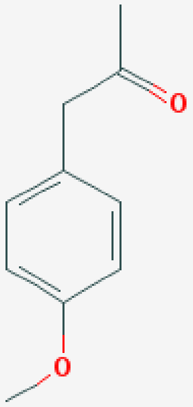	164.22	29.95	1.05	0.04
ANN	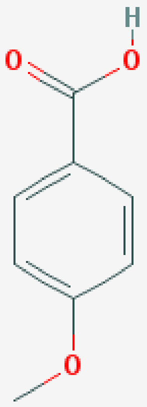	152.16	29.69	0.69	0.03

Molecule Name	Structure	MW	OB (%)	Caco-2	DL
APIOL	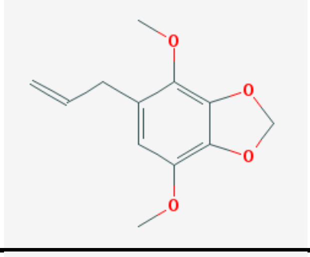	222.26	16.82	1.23	0.09
Arachic acid	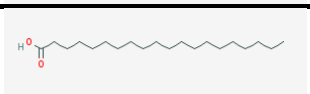	312.6	16.66	1.18	0.19
beta-sitosterol	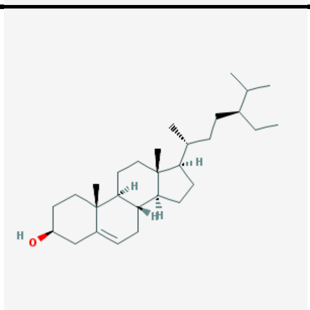	414.79	36.91	1.32	0.75
Butyrophenone	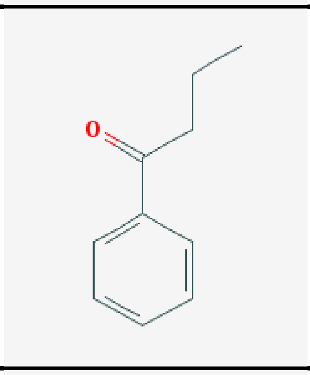	148.22	46.49	1.46	0.03
CAM	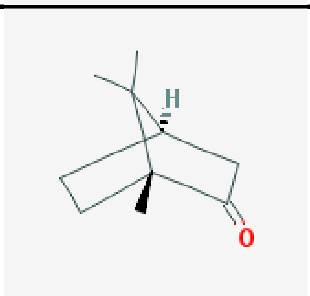	152.26	67.17	1.29	0.05
campesterol	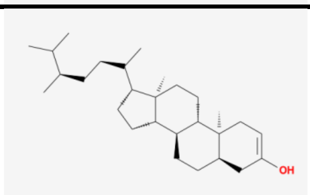	400.76	5.57	1.6	0.72

Molecule Name	Structure	MW	OB (%)	Caco-2	DL
cis-ligustilide	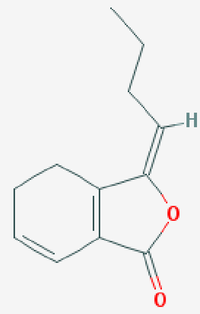	190.26	51.3	1.3	0.07
Cymol	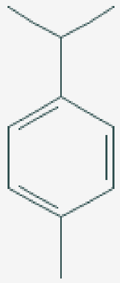	134.24	27.2	1.86	0.02
D-Camphene	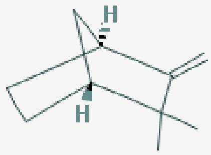	136.26	34.98	1.81	0.04
Docosanoate	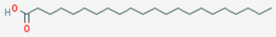	340.66	15.69	1.21	0.26
EIC	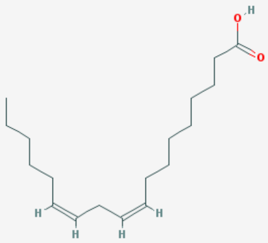	280.5	41.9	1.16	0.14
Fenchylacetate	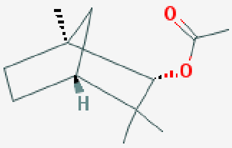	196.32	109.32	1.31	0.07

Molecule Name	Structure	MW	OB (%)	Caco-2	DL
Henicosanoic acid	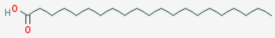	326.63	16.14	1.15	0.23
Isooleic acid	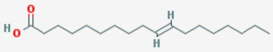	282.52	33.13	1.15	0.14
L-Limonen	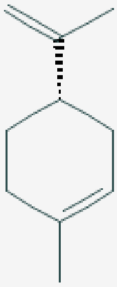	136.26	38.09	1.83	0.02
Majudin	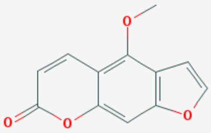	216.2	42.21	0.94	0.13
m-Cymol	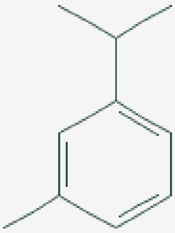	134.24	48.85	1.88	0.02
Moslene	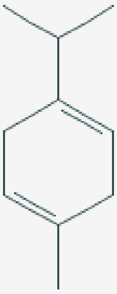	136.26	33.02	1.88	0.02

Molecule Name	Structure	MW	OB (%)	Caco-2	DL
Myrcene	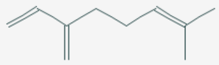	136.26	24.96	1.84	0.02
myristic acid	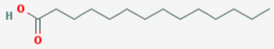	228.42	21.18	1.07	0.07
oleanolic acid	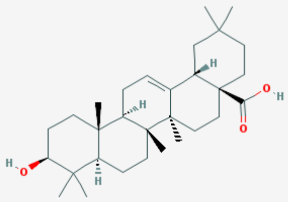	456.78	29.02	0.59	0.76
oleic acid	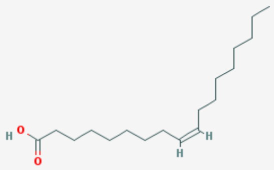	282.52	33.13	1.17	0.14
palmitic acid	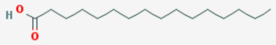	256.48	19.3	1.09	0.1
PENTADECYLIC ACID	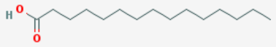	242.45	20.18	1.08	0.08
Petroselic acid	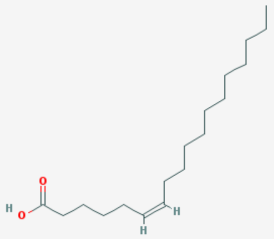	282.52	33.13	1.14	0.14
Sitogluside	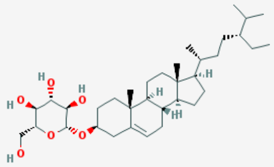	576.95	20.63	-0.14	0.62

Molecule Name	Structure	MW	OB (%)	Caco-2	DL
Skimmetin	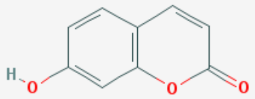	162.15	27.37	0.74	0.05
Stigmasterol	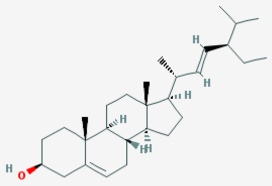	412.77	43.83	1.44	0.76
TDA	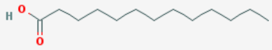	214.39	22.32	1.04	0.05
Terragon	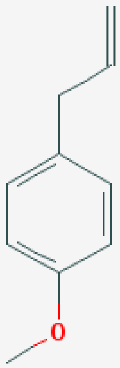	148.22	36.59	1.72	0.03
Uvadex	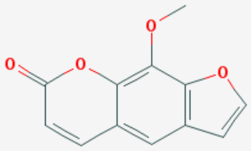	216.2	35.3	1.05	0.13
