# Supplementary material for: Analysis of Network Pharmacological Efficacy and Therapeutic Effectiveness in Animal Models for Functional Dyspepsia of Foeniculi fructus
Source: Nutrients. 2023 Jun 6;15(12):2644. doi: 10.3390/nu15122644 (PMC10301275; doi:10.3390/nu15122644)
Supplement: Supplementary file 1 [file nutrients-15-02644-s001.zip › Table S2 Target genes of Foeniculi fructus.pdf]

Supplementary Materials Table S2

Target genes of *Foeniculi Fructus*

Target name	Gene name	UniProt ID
2-hydroxy-6-oxo-7-methylocta-2,4-dienoate hydrolase	cmtE	Q51980
3-hydroxy-3-methylglutaryl-coenzyme A reductase	HMGCR	P04035
3-oxoacyl-[acyl-carrier-protein] synthase 1	kasA	P9WQD8
4-aminobutyrate aminotransferase, mitochondrial	ABAT	P80404
5-hydroxytryptamine 2A receptor	HTR2A	P28223
5-hydroxytryptamine receptor 3A	HTR3A	P46098
72 kDa type IV collagenase	MMP2	P08253
Acetylcholinesterase	ACHE	P22303
Acetyl-CoA acetyltransferase	ACAT1	P24752
Acyl-CoA desaturase	Scd1	P13516
Adenosylmethionine-8-amino-7-oxononanoate aminotransferase	bioA	P12995
Adiponectin	ADIPOQ	Q15848
ADP-ribosylation factor 1	ARF1	P84077
Alanine racemase	alr	Q8ZJ10
Alanyl-tRNA synthetase, cytoplasmic	AARS1	P49588
Alcohol dehydrogenase 1A	ADH1A	P07327
Alcohol dehydrogenase 1B	ADH1B	K7D361
Alcohol dehydrogenase 1C	ADH1C	P00326
Alcohol dehydrogenase 4	ADH4	P10127
Alcohol dehydrogenase class 4 mu/sigma chain	Adh7	A0A6P5PL15
Aldehyde dehydrogenase, dimeric NADP-preferring	ALDH3A1	P30838
Aldehyde dehydrogenase, mitochondrial	ALDH2	P05091
Aldose reductase	Aldose reductase	O15289
Alpha-1A adrenergic receptor	ADRA1A	P35348
Alpha-1B adrenergic receptor	ADRA1B	P35368
Alpha-1D adrenergic receptor	ADRA1D	P25100
Alpha-2A adrenergic receptor	ADRA2A	P08913
Alpha-2B adrenergic receptor	ADRA2B	P18089
Alpha-2C adrenergic receptor	ADRA2C	P18825
Alpha-ketoglutarate-dependent taurine dioxygenase	tauD	P37610
Amine oxidase [flavin-containing] A	MAOA	P21397
Amine oxidase [flavin-containing] B	Maob	Q8BW75
Angiotensinogen	AGT	P01019
Apoptosis regulator BAX	BAX	Q07812
Apoptosis regulator Bcl-2	BCL2	P10415

Target name	Gene name	UniProt ID
Arginase	ARG2	A0A024R6A0
Arginase-1	ARG1	P05089
Aromatic-amino-acid aminotransferase	tyrB	P04693
Aspartate aminotransferase	GOT1	P17174
Aspartyl aminopeptidase	DNPEP	Q9ULA0
ATP-dependent Clp protease ATP-binding subunit clpA	clpA	P0ABH9
Bacillolysin	npr	P23384
BDNF/NT-3 growth factors receptor	NTRK2	Q16620
Beta-1 adrenergic receptor	ADRB1	P08588
Beta-2 adrenergic receptor	ADRB2	P07550
Beta-amylase	BMY1	P10537
Beta-catenin	CTNNB1	P35222
Beta-galactosidase	GLB1	P16278
Beta-galactoside alpha-2,6-sialyltransferase 2	ST6GAL2	Q96JF0
Betaine--homocysteine S-methyltransferase 1	BHMT	Q93088
Beta-lactamase	ampC	P00811
Beta-lactamase SHV-1	SHV-1	A0A0H4IQA0
Beta-lactamase SHV-1 precursor	bla	P0AD63
Beta-lactamase SHV-2 precursor	bla	P0A9Z7
Biphenyl-2,3-diol 1,2-dioxygenase	bphC	P17297
Brain-derived neurotrophic factor	BDNF	A0A0E3SU01
Calcium-activated potassium channel subunit alpha 1	KCNMA1	Q12791
Calmodulin	CALM1	P0DP23
cAMP-dependent protein kinase inhibitor alpha	PKIA	P61925
Carbonic anhydrase II	CA2	P00918
Carboxylesterase 2	CES2	O00748
Caspase-3	CASP3	P42574
Caspase-8	CASP8	Q14790
Caspase-9	CASP9	P55211
Catalase	CAT	P04040
Cathepsin B	CTSB	P07858
Cathepsin D	CTSD	P07339
Cathepsin G	CTSG	P08311
Cbp/p300-interacting transactivator 1	CITED1	Q99966
CCAAT/enhancer-binding protein beta	CEBPB	P17676
Cell division protein kinase 4	CDK4	P11802
Cell-death-related nuclease 7	crn-7	crn-7
CGMP-inhibited 3',5'-cyclic phosphodiesterase A	PDE3A	Q14432
Cholecystokinin	CCK	P06307

Target name	Gene name	UniProt ID
Cholesteryl ester transfer protein	CETP	P11597
Choline-phosphate cytidylyltransferase A	PCYT1A	P49585
Cholinesterase	BCHE	P06276
Chymase	CMA1	P23946
Chymotrypsinogen B	CTRB1	P17538
Clavaminate synthase 1	cs1	Q05581
Coagulation factor VII	F7	P08709
Coagulation factor Xa	LOC113428980	A0A6J1VWA9
Collagen alpha-1(I) chain	COL1A1	P02452
Collagen alpha-1(VII) chain	COL7A1	Q02388
C-reactive protein	CRP	P02741
CUGBP Elav-like family member 1	CELF1	Q92879
Cyclic AMP-dependent transcription factor ATF-2	ATF2	P15336
Cysteine desulfurase	NFS1	Q9Y697
Cytochrome P450-cam	camC	P00183
D-alanyl-D-alanine carboxypeptidase	dac	P39045
Dihydroorotate dehydrogenase	pyrD	B2USR9
Dihydroorotate dehydrogenase, mitochondrial	DHODH	Q02127
Dipeptidyl peptidase IV	Dpp4	P28843
DNA polymerase (HSV)	DNA polymerase	Q308M6
DNA replication factor Cdt1	CDT1	Q9H211
DNA topoisomerase II	TOP2A	P11388
Dopamine D1 receptor	DRD1	P21728
Elastase 1	Elastase 1	Q7SIG3
Endothelin-1	EDN1	P05305
Fatty acid-binding protein, liver	FABP1	P07148
Ferrichrome-iron receptor	fhuA	O86925
Fibronectin	FN1	P02751
Formate acetyltransferase 1	pflB	P09373
G1/S-specific cyclin-D1	CCND1	P24385
Gag-Pol polyprotein	gag-pol	P03366
Gamma-aminobutyric acid receptor subunit alpha-1	GABRA1	P14867
Gamma-aminobutyric-acid receptor alpha-2 subunit	GABRA2	P47869
Gamma-aminobutyric-acid receptor alpha-3 subunit	GABRA3	P34903
Gamma-aminobutyric-acid receptor alpha-5 subunit	GABRA5	P31644
Gamma-aminobutyric-acid receptor subunit alpha-4	GABRA4	P48169
Gamma-aminobutyric-acid receptor subunit alpha-6	GABRA6	Q16445
Glucagon	GCG	P68274
Glucarate dehydratase	gudD	P0AES2

Target name	Gene name	UniProt ID
Glucose-6-phosphate isomerase	GPI	P06744
Glucose--fructose oxidoreductase	gfo	Q07982
Glutamate [NMDA] receptor subunit 3A	GRIN3A	Q8TCU5
Glutamate [NMDA] receptor subunit epsilon 1	NMDE1	U6D0N7
Glutamate receptor 2	GRIA2	P42262
Glutamyl aminopeptidase	ENPEP	Q07075
Glycine amidinotransferase, mitochondrial	GATM	P50440
Glycine N-methyltransferase	GNMT	Q14749
Glycine receptor alpha-1 chain	GLRA1	P23415
Glycine receptor subunit alpha-2	GLRA2	P23416
Glycine receptor subunit alpha-3	GLRA3	O75311
Group IIE secretory phospholipase A2	PLA2G2E	Q9NZK7
Guanidinoacetate N-methyltransferase	GAMT	Q14353
Gyrase B	gyrB	P0AES6
Haloalkane dehalogenase	dhaA	P0A3G2
Heat shock protein HSP 90	HSP90AB1	P08238
Heme oxygenase 1	HMOX1	P09601
Histidinol dehydrogenase	hisD	P06988
Ig gamma-1 chain C region	IGHG1	P01857
Insulin	INS	P01308
Intercellular adhesion molecule 1	ICAM1	K7EKL8
Interleukin-1 beta	IL1B	P01584
Interleukin-10	IL10	P22301
Interleukin-2	IL2	P60568
Interleukin-6	IL6	P05231
Interstitial collagenase	MMP1	P03956
Kappa-type opioid receptor	OPRK1	E5RJI5
Kynureninase	KYNU	Q16719
Leukotriene A-4 hydrolase	LTA4H	P09960
Lipoprotein lipase	LPL	Q6IAV0
Lysozyme	LYZL1	H0YDZ2
Macrophage metalloelastase	Mmp12	P34960
Malonamidase E2	AZC_1059	A8HQS6
Mannose-6-phosphate isomerase	MPI	P34949
Matrix metalloproteinase-9	MMP9	P14780
Metalloproteinase inhibitor 1	TIMP1	P01033
Metalloproteinase inhibitor 2	TIMP2	P16035
Metallothionein-2	MT2A	P02795
Microtubule-associated protein 2	MAP2	P11137

Target name	Gene name	UniProt ID
Mineralocorticoid receptor	NR3C2	P08235
Mitochondrial uncoupling protein 2	UCP2	P55851
Mitochondrial uncoupling protein 3	UCP3	P55916
Mitogen-activated protein kinase 1	Mapk1	P63085
Mitogen-activated protein kinase 10	MAPK10	P53779
Mitogen-activated protein kinase 8	MAPK8	P45983
Mitogen-activated protein kinase 9	MAPK9	P45984
M-phase inducer phosphatase 2	CDC25B	P30305
mRNA of PKA Catalytic Subunit C-alpha	PRKACA	P17612
mRNA of Protein-tyrosine phosphatase, non-receptor type 1	PTPN1	P18031
Muscarinic acetylcholine receptor M1	CHRM1	P11229
Muscarinic acetylcholine receptor M2	CHRM2	P08172
Muscarinic acetylcholine receptor M3	CHRM3	P20309
Muscarinic acetylcholine receptor M4	CHRM4	P08173
Mu-type opioid receptor	OPRM1	P35372
Myc proto-oncogene protein	MYC	P01106
Myeloperoxidase	MPO	P05164
NAD(P)H dehydrogenase [quinone] 1	NQO1	P15559
NAD-dependent malic enzyme, mitochondrial	ME2	P23368
NADP-dependent malic enzyme, mitochondrial	ME3	Q16798
NADPH-ferredoxin reductase fprA	fprA	P9WIQ3
Neuromodulin	GAP43	P17677
Neuronal acetylcholine receptor protein, alpha-7 chain	CHRNA7	P36544
Neuronal acetylcholine receptor subunit alpha-2	CHRNA2	Q15822
Neuronal acetylcholine receptor subunit alpha-4	CHRNA4	P43681
NF-kappa-B inhibitor alpha	NFKBIA	P25963
Nicotinate-nucleotide--dimethylbenzimidazole phosphoribosyltransferase	cobT	F0L709
Nitric oxide synthase, inducible	NOS2	P35228
Nitric-oxide synthase, brain	NOS1	P29475
Nitric-oxide synthase, endothelial	NOS3	P29474
NRH dehydrogenase [quinone] 2	NQO2	P16083
Nuclear receptor coactivator 1	NCOA1	Q15788
Nuclear receptor coactivator 2	NCOA2	Q15596
Oligopeptide ABC transporter, periplasmic oligopeptide-binding protein	oppA	B1LH57
Oxalate decarboxylase oxdC	oxdC	O34714
Pancreas/duodenum homeobox protein 1	PDX1	P52945
Pancreatic alpha-amylase	AMY2A	P04746

Target name	Gene name	UniProt ID
Parathion hydrolase	opd	P0A434
Peptide YY	Pyy	H3BK86
Peptidyl-glycine alpha-amidating monooxygenase	PAM	P19021
Peripheral plasma membrane protein CASK	CASK	O14936
Peroxisome proliferator-activated receptor alpha	PPARA	Q07869
Peroxisome proliferator-activated receptor delta	PPARD	Q03181
Peroxisome proliferator-activated receptor gamma	PPARG	P37231
Phenylethanolamine N-methyltransferase	PNMT	P11086
Phosphatidylcholine-sterol acyltransferase	LCAT	P04180
Phosphatidylinositol-3,4,5-trisphosphate 3-phosphatase and dual-specificity protein phosphatase PTEN	PTEN	F6KD01
Phosphatidylinositol-4,5-bisphosphate 3-kinase catalytic subunit, gamma isoform	PIK3CG	P48736
Phospholipase A2	PLA2G1B	P04054
Phosphomannomutase 2	PMM2	O15305
Phosphoribosylglycinamide formyltransferase 2	purT	E6PVU1
Phosphotriesterase	opdA	Q93LD7
Plasminogen	PLG	P00747
Plasminogen activator inhibitor 1	SERPINE1	P05121
Potassium voltage-gated channel subfamily H member 2	Kcnh2	O35219
Proenkephalin-A	PENK	P01210
Progesterone receptor	PGR	P06401
Proline dehydrogenase, mitochondrial	PUT1	P09368
Pro-opiomelanocortin	POMC	P01189
Prostaglandin G/H synthase 1	PTGS1	P23219
Prostaglandin G/H synthase 2	PTGS2	P35354
Protein kinase C alpha type	PRKCA	P17252
Protein kinase C beta type	PRKCB	P05771
Proto-oncogene c-Fos	FOS	P01100
P-selectin	SELP	P16109
Purine nucleoside phosphorylase	PNP	P00491
Putative beta-glucuronidase-like protein SMA3	GUSBP1	Q15486
Putative cytochrome P450 130	cyp130	P9WPN4
Pyrroline-5-carboxylate reductase 1	PYCR1	P32322
Pyruvate dehydrogenase E1 component subunit beta, mitochondrial	PDHB	P11177
Quinoprotein glucose dehydrogenase-B	gdhB	P13650
Receptor tyrosine-protein kinase erbB-2	ERBB2	P04626
Renin, renal	REN	P00797

Target name	Gene name	UniProt ID
Retinoic acid receptor beta	RARB	P10826
Retinoic acid receptor RXR-alpha	RXRA	P19793
Retinol-binding protein 2	RBP2	P50120
Rhinovirus coat protein	Rhinovirus coat protein	Q82122
Rhodopsin	RHO	P08100
Ribonucleoside-diphosphate reductase large subunit	RRM1	P23921
Ryanodine receptor 2	RYR2	Q92736
Serum paraoxonase/arylesterase 1	PON1	P27169
Sigma factor sigB regulation protein rsbQ	L195_g027591	A0A2K3KZN0
Sodium channel protein type 5 subunit alpha	SCN5A	Q14524
Sodium-dependent dopamine transporter	SLC6A3	Q01959
Sodium-dependent noradrenaline transporter	SLC6A2	P23975
Sodium-dependent serotonin transporter	SLC6A4	P31645
Solute carrier family 2, facilitated glucose transporter member 2	SLC2A2	P11168
Solute carrier family 22 member 5	SLC22A5	O76082
Sterol O-acyltransferase 1	SOAT1	P35610
Streptavidin	Streptavidin	P22629
Stromelysin-1	MMP3	P08254
Subtilisin BPN'	apr	P00782
Subtilisin Carlsberg	subC	P00780
Superoxide dismutase [Cu-Zn]	SOD1	P00441
Telomerase protein component 1	TEP1	Q99973
Thioredoxin reductase, cytoplasmic	TXNRD1	Q16881
Thrombin	thrombin	Q28731
Transcription factor AP-1	JUN	P05412
Transcription factor p65	RELA	A0A087X0W8
Transforming growth factor beta-1	TGFB1	P01137
Transient receptor potential cation channel subfamily V member 1	TRPV1	Q8NER1
Triosephosphate isomerase	TPI1	P60174
Trypsin-3	PRSS3	P35030
Tumor necrosis factor	TNF	P01375
Tyrosinase	TYR	L8B082
Urokinase-type plasminogen activator	PLAU	S4R3G7
Xanthine dehydrogenase/oxidase	XDH	P47989
Zymogen granule membrane protein 16	ZG16	O60844
