# Supplementary material for: Analysis of Network Pharmacological Efficacy and Therapeutic Effectiveness in Animal Models for Functional Dyspepsia of Foeniculi fructus
Source: Nutrients. 2023 Jun 6;15(12):2644. doi: 10.3390/nu15122644 (PMC10301275; doi:10.3390/nu15122644)
Supplement: Supplementary file 1 [file nutrients-15-02644-s001.zip › Table S3 100 functional dyspepsia–related genes.pdf]

Supplementary Materials Table S3

One hundred functional dyspepsia–related genes

Gene name	Protein name
ACHE	Acetylcholinesterase (Yt blood group)
ADRA2A	Alpha-2 adrenergic receptor subtype C10
ATP12A	ATPase, H+/K+ transporting, nongastric, alpha polypeptide
ATP4A	ATPase, H+/K+ exchanging, alpha polypeptide
BCHE	Acylcholine acylhydrolase
BDNF	Brain-derived neurotrophic factor
BHLHE22	Trinucleotide repeat-containing gene 20 protein
CALCA	Calcitonin-related polypeptide alpha
CCK	Cholecystokinin
CCKAR	Cholecystokinin receptor type A
CCL28	Mucosae-associated epithelial chemokine
CD4	T-cell surface antigen T4/Leu-3
CELA1	Chymotrypsin-like elastase family, member 1
CELA3B	Chymotrypsin-like elastase family, member 3B
CHGA	Chromogranin A (parathyroid secretory protein 1)
CLDN3	Clostridium perfringens enterotoxin receptor 2
COMT	Catechol O-methyltransferase
CRH	Corticotropin releasing hormone
CRHR1	Corticotropin releasing hormone receptor 1
CRHR2	Corticotropin releasing hormone receptor 2
CRP	C-reactive protein, pentraxin-related
CXCL8	Monocyte-derived neutrophil chemotactic factor
CYBRD1	Ferric-chelate reductase 3
CYP2C19	Cytochrome P450, family 2, subfamily C, polypeptide 19
DEFB4B	Skin-antimicrobial peptide 1
DRD2	D(2) dopamine receptor
FOS	FBJ murine osteosarcoma viral oncogene homolog
GAST	Gastrin
GCG	Glucagon
GHRL	Growth hormone-releasing peptide
GHSR	Growth hormone secretagogue receptor type 1
GIP	Glucose-dependent insulinotropic polypeptide
GNB3	Guanine nucleotide binding protein (G protein), beta polypeptide 3
GPBAR1	G protein-coupled bile acid receptor 1

Gene name	Protein name
GPT	Glutamic-pyruvate transaminase (alanine aminotransferase)
HR	Lysine-specific demethylase hairless
HRH2	Histamine H2 receptor
HTR1A	5-hydroxytryptamine (serotonin) receptor 1A, G protein-coupled
HTR1B	5-hydroxytryptamine (serotonin) receptor 1B, G protein-coupled
HTR1D	5-hydroxytryptamine (serotonin) receptor 1D, G protein-coupled
HTR2B	5-hydroxytryptamine (serotonin) receptor 2B, G protein-coupled
HTR2C	5-hydroxytryptamine (serotonin) receptor 2C, G protein-coupled
HTR3A	5-hydroxytryptamine (serotonin) receptor 3A, ionotropic
HTR3B	5-hydroxytryptamine (serotonin) receptor 3B, ionotropic
HTR3C	5-hydroxytryptamine (serotonin) receptor 3C, ionotropic
HTR3D	5-hydroxytryptamine (serotonin) receptor 3D, ionotropic
HTR3E	5-hydroxytryptamine (serotonin) receptor 3E, ionotropic
HTR4	5-hydroxytryptamine (serotonin) receptor 4, G protein-coupled
HTR7	5-hydroxytryptamine (serotonin) receptor 7, adenylate cyclase-coupled
IL10	Cytokine synthesis inhibitory factor
IL17F	Interleukin 17F
IL1B	Interleukin 1, beta
IL6	B-cell stimulatory factor 2
INS	Insulin
JUN	V-jun avian sarcoma virus 17 oncogene homolog
KCNH2	Potassium voltage-gated channel, subfamily H (eag-related), member 2
LEP	Obesity factor
LIPN	Lipase-like abhydrolase domain-containing protein 4
LRP5	Low density lipoprotein receptor-related protein 5
MBOAT4	Membrane-bound O-acyltransferase domain-containing protein 4
MLN	Promotilin
MLNR	G-protein coupled receptor 38
MTRNR2L5	MT-RNR2-like protein 5
NDNF	Neuron-derived neurotrophic factor
NGF	Nerve growth factor (beta polypeptide)
NLRP9	Nucleotide-binding oligomerization domain protein 6
NOS1	Peptidyl-cysteine S-nitrosylase NOS1
NPR3	Atrial natriuretic peptide clearance receptor
NPY	Pro-neuropeptide Y
OCLN	Occludin
PCSK5	Proprotein convertase subtilisin/kexin type 5

Gene name	Protein name
PKD1L3	Polycystic kidney disease protein 1-like 3
PMM1	Phosphomannomutase 1
POMC	Corticotropin-lipotropin
PPY	Pancreatic polypeptide
PRL	Prolactin
PTGS1	Prostaglandin-endoperoxide synthase 1 (prostaglandin G/H synthase and cyclooxygenase)
PTGS2	Prostaglandin-endoperoxide synthase 2 (prostaglandin G/H synthase and cyclooxygenase)
PYY	Peptide tyrosine tyrosine
RNASE2	Ribonuclease, RNase A family, 2 (liver, eosinophil-derived neurotoxin)
RNASE3	Ribonuclease, RNase A family, 3
S100A8	Migration inhibitory factor-related protein 8
SCN10A	Sodium channel, voltage-gated, type X, alpha subunit
SLC6A4	Solute carrier family 6 (neurotransmitter transporter), member 4
SP9	Sp9 transcription factor
SRSF10	FUS-interacting serine-arginine-rich protein 1
SST	Growth hormone release-inhibiting factor
TAC1	Tachykinin, precursor 1
TGM2	Protein-glutamine gamma-glutamyltransferase 2
TJP1	Tight junction protein ZO-1
TLR2	Toll/interleukin-1 receptor-like protein 4
TNF	Tumor necrosis factor ligand superfamily member 2
TPH1	Tryptophan hydroxylase 1
TRPA1	Transient receptor potential cation channel, subfamily A, member 1
TRPM5	Transient receptor potential cation channel, subfamily M, member 5
TRPM8	Transient receptor potential cation channel, subfamily M, member 8
TRPV2	Transient receptor potential cation channel, subfamily V, member 2
TSTD2	Thiosulfate sulfurtransferase/rhodanese-like domain-containing protein 2
VIP	Vasoactive intestinal peptide
VR1	Transient receptor potential cation channel, subfamily V, member 1
